# Case Report: Omalizumab combined with voriconazole for the treatment of ABPA complicating IPA: a case report

**DOI:** 10.3389/fphar.2025.1588182

**Published:** 2025-07-02

**Authors:** Tingrui Zhao, Yukai Chen, He Sun

**Affiliations:** ^1^ Department of Clinical Pharmacy, The Third Hospital of Mianyang, Sichuan Mental Health Center, Mianyang, China; ^2^ Department of Respiratory and Critical Care Medicine, Shanghai East Hospital, Tongji University School of Medicine, Shanghai, China

**Keywords:** allergic bronchopulmonary aspergillosis, glucocorticoids, omalizumab α, dupilumab, voriconazole

## Abstract

Aspergillus invading hosts may manifest as Allergic bronchopulmonary aspergillosis (ABPA) or invasive pulmonary aspergillosis (IPA) in individuals with varying immune statuses. ABPA predominantly occurs in severe asthma patients, whereas IPA is typically observed in immunocompromised individuals. ABPA management centers on glucocorticoids to mitigate hypersensitivity-driven inflammation, while IPA requires aggressive antifungal therapy. Concurrent ABPA and IPA presents a therapeutic dilemma, as glucocorticoids use may exacerbate fungal dissemination, while antifungal agents alone inadequately address the allergic component. Adjusting treatment strategies to balance immunosuppression to control ABPA with sufficient antifungal coverage for IPA is critical step. The case report presents an innovative therapeutic strategy for a 73-year-old female with co-existing ABPA and IPA. After suboptimal clinical response to conventional glucocorticoid-antifungal therapy, we implemented a guideline-aligned, evidence-based regimen combining omalizumab with voriconazole. While this dual therapy achieved clinical stabilization, persistently elevated serum IgE (>5000 IU/mL). By reviewing the literature and comparing the differences between the mechanisms of omalizumab and dupilumab, the treatment was finally changed from omalizumab to dupilumab and followed up. This case is also a practice guided by ISHAM guidelines while pioneering a mechanism-driven transition from omalizumab to dupilumab in ABPA-IPA co-management. In order to provide guidance for the treatment of ABPA-IPA disease.

## Introduction

Allergic Bronchopulmonary Aspergillosis (ABPA) is a complex pulmonary disorder primarily affecting patients with asthma or cystic fibrosis, characterized by an exaggerated allergic response to Aspergillus species such as Aspergillus fumigatus (Maturu and Agarwal, 2015a; Agarwal et al., 2024). Untreated chronic progression of ABPA may result in bronchiectasis and pulmonary fibrosis. Standard treatment relies on glucocorticoids combined with antifungal agents to suppress allergic reactions and reduce fungal burden (Agarwal et al., 2024).

In contrast, Invasive Pulmonary Aspergillosis (IPA) predominantly occurs in immunocompromised hosts, manifesting as tissue-invasive fungal infection and requiring early aggressive antifungal therapy (e.g., voriconazole, isavuconazole and amphotericin B) (Ullmann et al., 2018; Garcia-Vidal et al., 2019). The co-occurrence of ABPA and IPA presents a unique therapeutic dilemma: immunosuppression required for ABPA control risks exacerbating IPA progression, while antifungal monotherapy inadequately addresses the allergic cascade of ABPA. This imbalance in immune regulation poses significant clinical challenges.

Notably, targeted biologic therapies have recently emerged as promising strategies to resolve this therapeutic paradox. Omalizumab α, an anti-IgE monoclonal antibody, disrupts the allergic cascade in ABPA by blocking IgE binding to the FcεRI receptor on effector cells (Agarwal et al., 2024). Its mechanism of action offers dual advantages—reducing glucocorticoids dependence while preserving innate antifungal immunity by avoiding broad-spectrum immunosuppression.

Building on the mechanistic advantages of biologic agents, we present a case where combined omalizumab α and voriconazole therapy resolved this complex interplay, followed by a mechanism-driven transition to dupilumab for refractory IgE elevation, providing new insights into resolving such immunotherapy conflicts.

## Case description

The patient, a 73-year-old female, was admitted to the hospital for “recurrent cough, expectoration, and wheezing for over 20 years, with exacerbation over the past month.” She has a history of “chronic obstructive pulmonary disease (COPD), asthma, and bronchiectasis.” Auscultation of both lungs revealed diminished breath sounds with a notably prolonged expiratory phase, and low-pitched wheezing can be heard in both lungs.

The patient had prolonged symptoms of chest tightness and wheezing, and had been using prednisone tablets (10–30 mg daily) irregularly for a long time (>1 year), but still had symptoms of dyspnea. She had also received intravenous steroid therapy several times during acute exacerbation.

**FIGURE 1:**
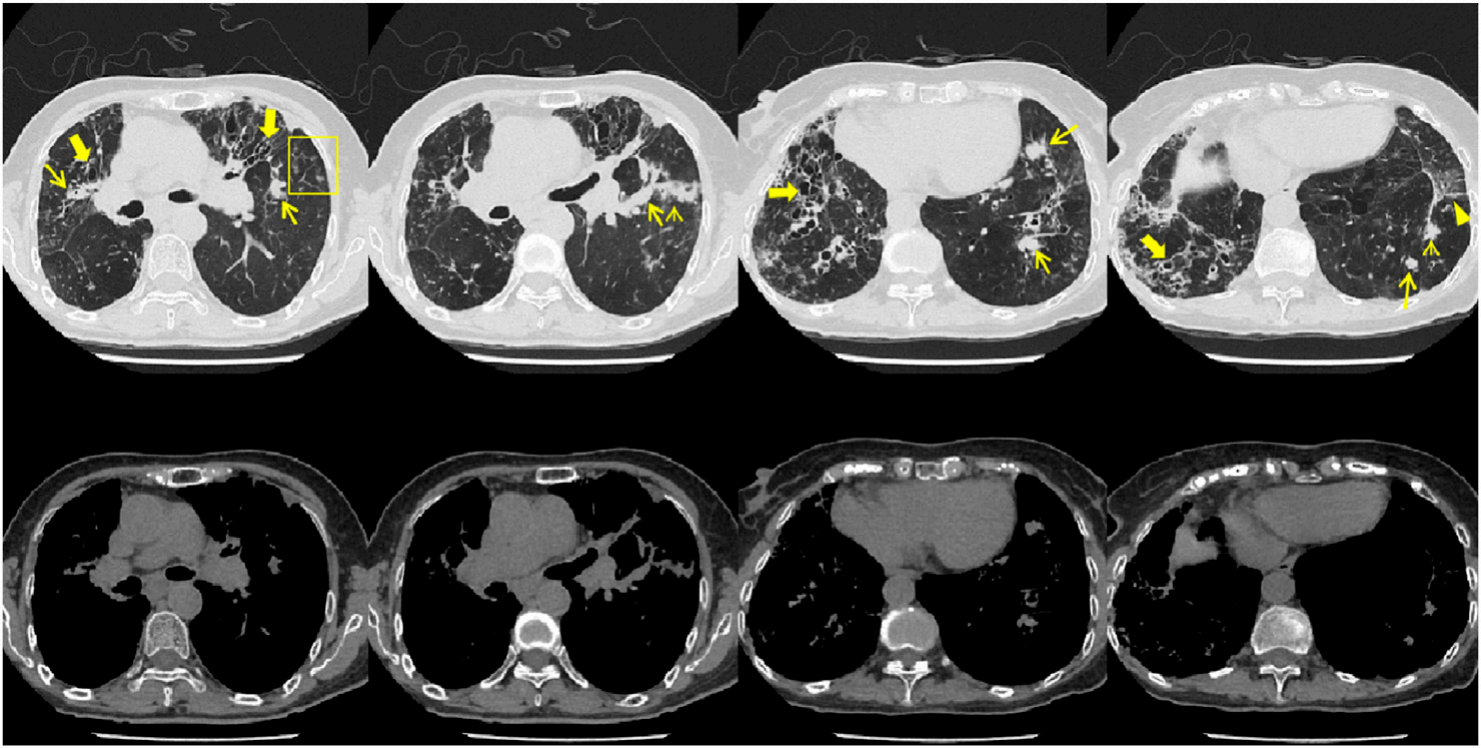
Patient’s chest CT imaging on 14 Sep 2023. The chest computed tomography showed bronchiectasis (bold arrows), mucus pluggings (narrow arrows) and chronic pleuropulmonary fibrosis (triangles) before treatment, which were typical manifestations of ABPA. Bronchopneumonia (arrow heads) and tree-in-bud opacities (yellow boxes) were found, indicating cocurrent IPA.

In July 2023, the patient visited the local hospital for “chest pain, cough, and sputum”, and the results of chest CT showed multiple bronchiectasis with mucus plugs in both lungs (Figure 1), but no antifungal treatment was administered. The patient was readmitted to our hospital on 18 September 2023, with exacerbated symptoms of chest tightness and wheezing. Laboratory results showed white blood cell count (10.32 × 10^9^/L) and the absolute eosinophil count (3.84 × 10^9^/L) (Table 1), the Bronchoalveolar Lavage Fluid (BALF) culture was positive for Aspergillus fumigatus, and the sputum culture was positive for *Candida* albicans. The peripheral blood Glactomannan test (GM) was negative. A transbronchial lung biopsy suggested acute and chronic inflammation of the bronchial mucosa, with mucus plug formation, was diagnosed with acute exacerbation of chronic obstructive pulmonary disease, with bronchodilation and concurrent infection. Itraconazole capsule was prescribed for oral treatment (200 mg qd for 1 year). During outpatient treatment phase, the patient experienced recurrent symptoms accompanied by intermittent febrile episodes.

**TABLE 1:** Result of peripheral blood inflammation markers.

	09.18.2023	05.04.2024	05.26.2024	05.29.2024	09.09.2024
WBC (×10^9^/L)	10.32	16.86	10.11	6.94	8.96
NR (%)	41	80.2	86.7	64.5	72.3
PCT (ng/mL)	0.053	0.051	0.248	0.18	<0.02
CRP (mg/L)	8.07	146.65	49.89	11.74	<1.6
ESR (mmh)	97	120	63	55	50
AEC (×10^9^/L)	3.84	0.68	0.34	0.3	0.15
Serum IgE (IU/mL)	>2500	>2500	>2500	>2500	>5000

WBC, white blood cell count; NR, neutrophil ratio; PCT, procalcitonin; CRP, C-reactive protein; ESR, erythrocyte sedimentation Rate; AEC, absolute eosinophil count; Serum IgE, Serum Immunoglobulin E.

On 4 May 2024, the patient was readmitted to the hospital, and the following examination results were recored: blood C-reactive protein (146 mg/L), erythrocyte sedimentation rate (120 mm/h), white blood cell count (16.86 × 10 ^ 9/L), absolute eosinophil count (0.68 × 10 ^ 9/L), procalcitonin (0.051 ng/mL), CD4^+^ count 628/μL, CD8^+^ count 354/μL and total serum Immunoglobulin E (IgE) (>2500 U/mL) (Table 1), HIV seronegative, BALF target NGS (tNGS) showed *Haemophilus* influenzae (1 × 10 ^ 4 copies/mL), Aspergillus fumigatus (<1 × 10 ^ 2 copies/mL), Epstein-Barr virus (EBV), and rhinovirus. The two separate BALF GM tests yielded results of 2.1 μg/L and 2.71 μg/L, respectively.

The diagnosis proposed was: community-acquired pneumonia (Aspergillus fumigatus in combination with Hemophilus influenzae), though ABPA was not excluded. The patient was instructed to discontinue oral steroid therapy and was treated with omalizumab α (Mabpharm Limitied) (300 mg biweekly subcutaneous injection), voriconazole (Huahai Pharmaceutical Co., Ltd.) (200 mg q12h po), and nemonoxacin (0.5g IV qd). The patient’s symptoms significantly improved, and she was discharged from the hospital. Asthma symptom assessment indicated a cough severity score of 13 on the Cough Evaluation Test (CET), an Asthma Control Questionnaire (ACT) score of 17, and an Asthma Control Questionnaire-5 (ACQ-5) score of 2.8. Pulmonary function showed severe obstructive ventilatory dysfunction, with a positive bronchodilator test (Table 2).

**TABLE 2:** Changes in asthma control symptom scores and lung function before and after treatment with omalizumab α in combination with voriconazole.

	05.2024	08.2024
CET	13	7
ACT	17	25
ACQ-5	2.8	0.8

CET, cough evaluation test; ACT, asthma control questionnaire; ACQ-5, Asthma Control Questionnaire-5.

**FIGURE 2:**
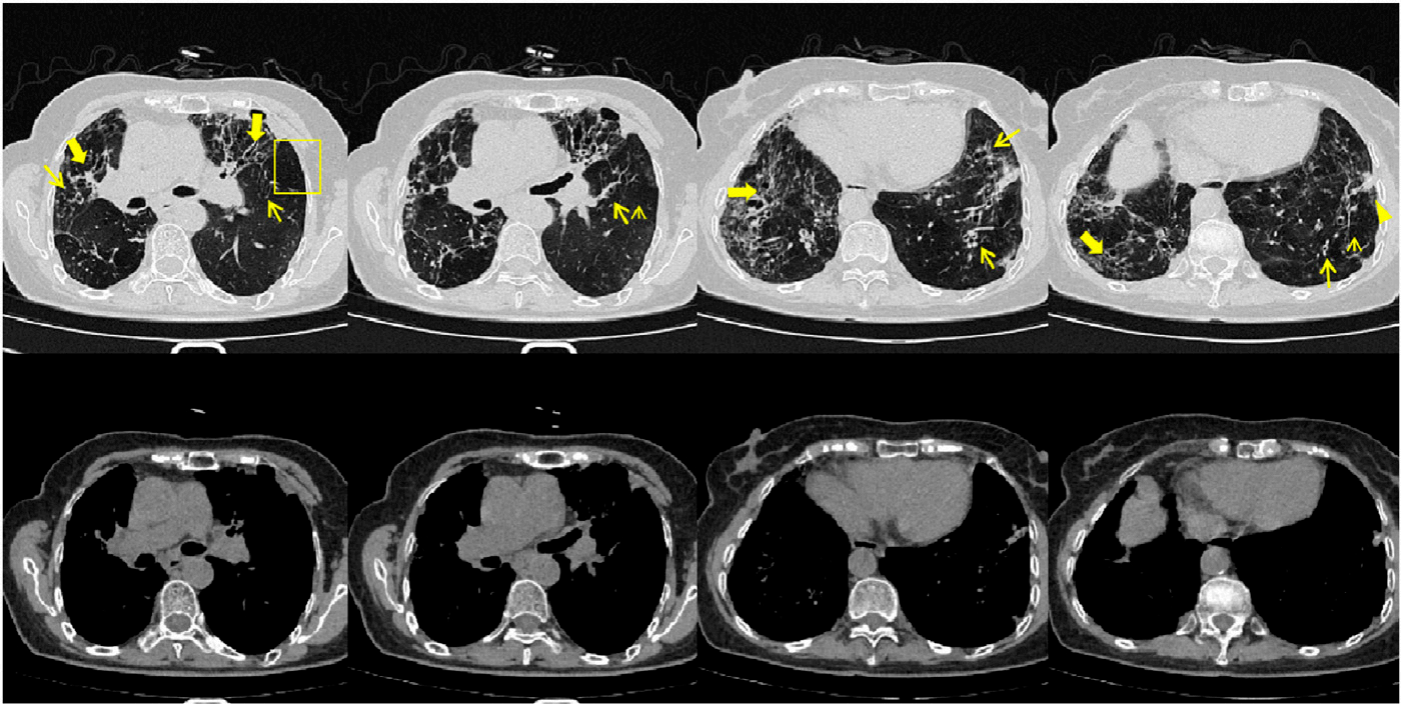
Patient’s chest CT imaging on 28 May 2024. The chest computed tomography showed bronchiectasis (bold arrows) and chronic pleuropulmonary fibrosis (triangles), with remission of mucus plugging (narrow arrows), bronchopneumonia (arrow heads) and tree-in-bud opacities (yellow boxes) compared to Figure 1, indicating partial remission of ABPA and IPA.

**FIGURE 3:**
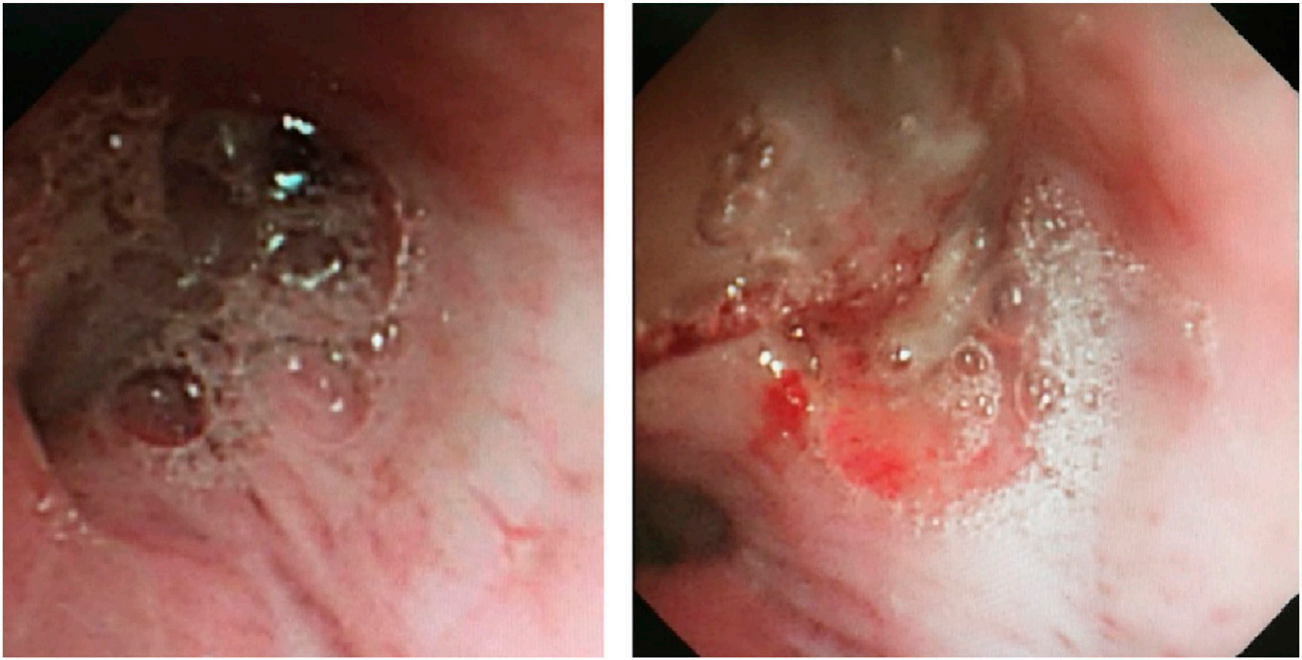
Fiberoptic bronchoscopy findings. Fiberoptic bronchoscopy view of bilateral bronchial lumens seen with large amounts of purulent mucus secretions and mucus plugs filling the anterior portion of the left upper lobe.

**FIGURE 4:**
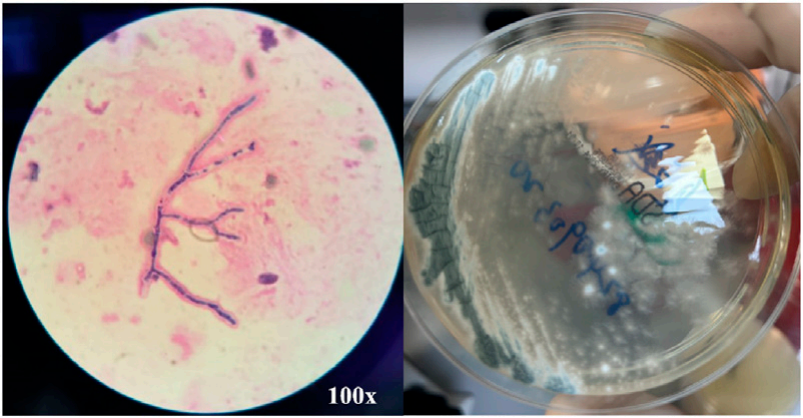
BALF Smear Staining and Culture Results. BALF Smear Regi Staining shows 45° branching with septate hyphae (left) and culture shows gray-green colonies (right), consistent with Aspergillus fumigatus.

On 26 May 2024, the patient returned to our hospital for further treatment. Chest CT showed multiple bronchiectasis with infection in both lungs and thickening of the bilateral pleura (Figure 2). Laboratory results showed white blood cell count (10.11 × 10^9^/L), absolute eosinophil count (0.34 × 10^9^/L), C-reactive protein (49.89 mg/L), procalcitonin (0.248 ng/mL), and total serum IgE (>2500 U/mL). Fiberoptic bronchoscopy revealed large amounts of jelly-like purulent mucus in the bilateral bronchial lumens, with bronchial mucosa edema and narrowed lumens. In the left upper lobe anterior segmental bronchus, thick jelly-like purulent mucus secretions were seen obstructing the passage. After repeated flushing and suctioning of the mucus plugs, the distal bronchus was patent, and no neoplasms or bleeding were observed (Figure 3). Cuentrifgal precipitate smear of BALF was stained with Raget’s stain, and 45-degree branched septate hyphae were seen under the microscope, and gray-green colonies were seen in the culture, which were consistent with Aspergillus fumigatus (Figure 4). The BALF GM test was 0.13 μg/L, and the tNGS of the BALF reported Aspergillus flavus (1 × 10^2^ copies/mL) and EBV. A. fumigatus-specific IgE was 44.4 kUA·L^−1^.

This patient was diagnosed with APBA complicated by IPA. The patient continued treatment with omalizumab α (300 mg biweekly subcutaneous injection), voriconazole (200 mg q12h po), and budesonide/formoterol inhalation powder (320/9 µg inhaled bid). The patient’s symptoms improved significantly.

**FIGURE 5:**
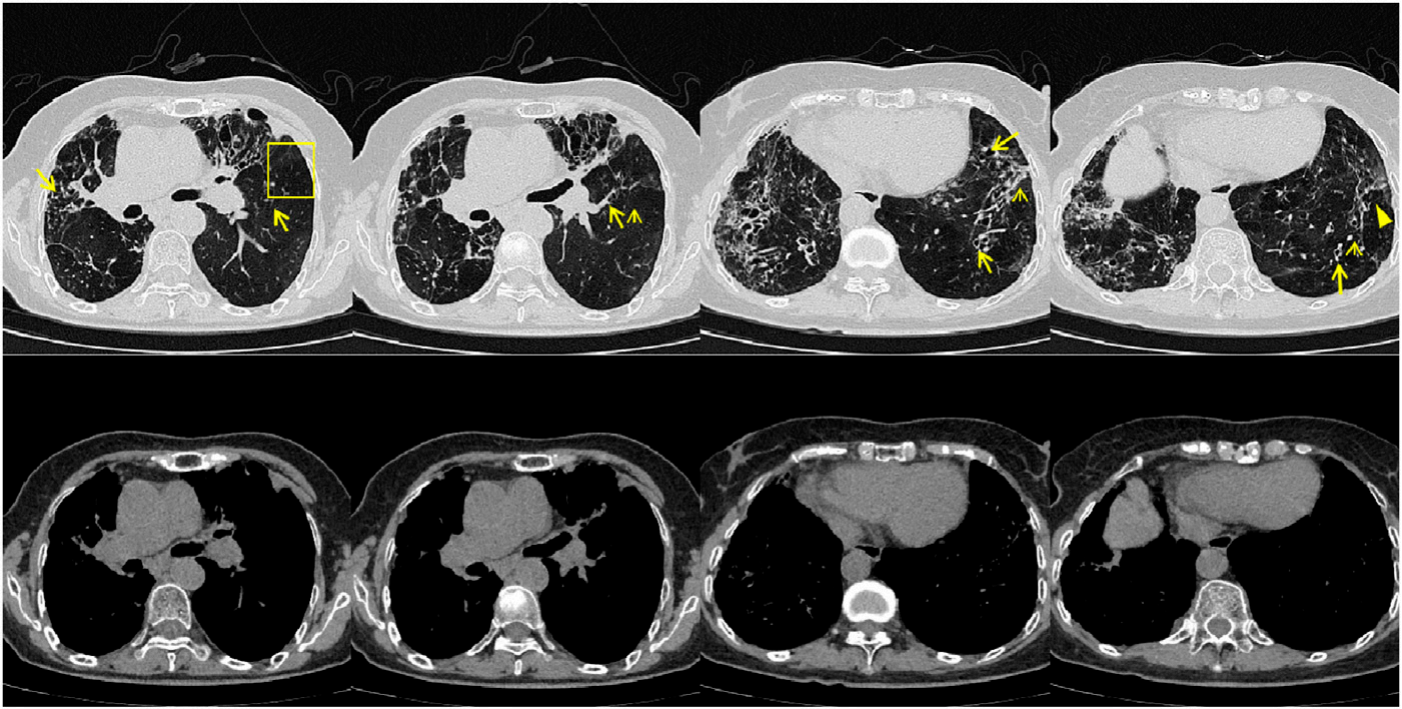
Patient’s chest CT imaging on Jun 17, 2024. The chest computed tomography showed remission of mucus plugging (narrow arrows), bronchopneumonia (arrow heads) and tree-in-bud opacities (yellow boxes) than previous images, indicating that the treatment was effective.

**FIGURE 6:**
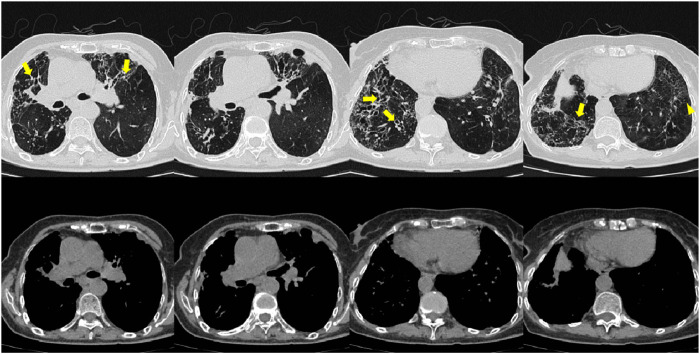
Patient’s chest CT imaging on Sep 12, 2024. The chest computed tomography showed stable disease of bronchiectasis (bold arrows) and chronic pleuropulmonary fibrosis (triangles), indicating the disease was consistently under control.

From June to September 2024, the patient followed up at the outpatient clinic. Chest CT showed that the lesions in both lungs were partially absorbed (Figures 5, 6), inflammatory markers returned to normal (Table 1), FeNO and peripheral blood eosinophils significantly decreased (Table 1, 3). By asthma symptom assessment, the patient’s cough severity score on the Cough Evaluation Test (CET) was 7, the Asthma Control Questionnaire (ACT) score was 25, and the Asthma Control Questionnaire-5 (ACQ-5) score was 0.8, indicating a significant improvement in pulmonary ventilation function (Table 2).

**TABLE 3:** Results of lung function before and after treatment with omalizumab α in combination with voriconazole.

	05.2024	08.2024
FeNO (ppb)	45	22
FEV1/FVC	46%	74.6%
FEV1%	37.6%	67%
PEF	17%	31.7%

FeNO, fractional exhaled nitric oxide; FEV1, forced expiratory volume in one second; FVC, forced vital capacity; FEV1%, Forced Expiratory Volume in one second percent; PEF, peak expiratory flow.

**FIGURE 7:**
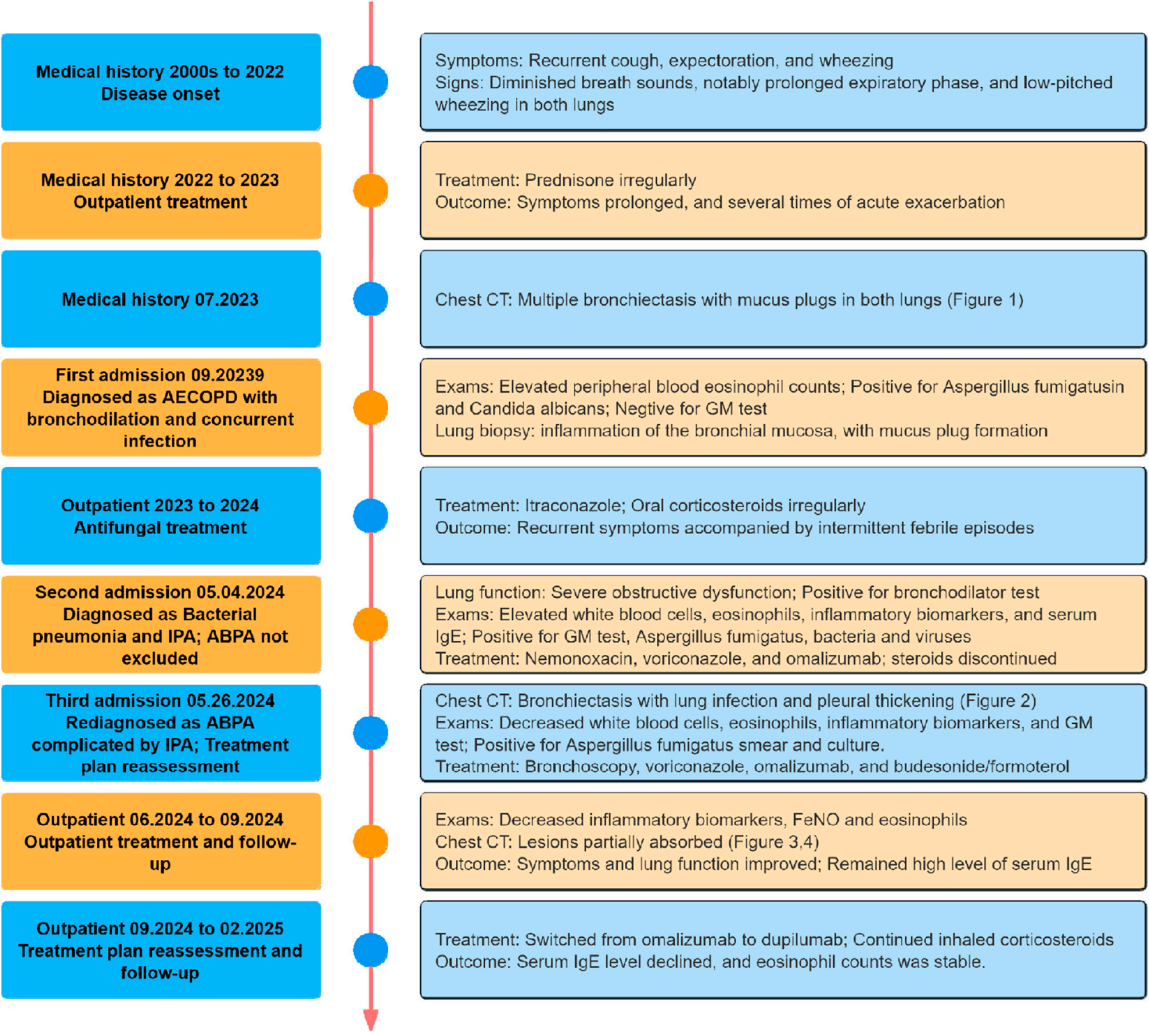
Timeline of the case treatment procedure. Abbreviations: CT, Computed Tomography; GM, Galactomannan; IgE, Immunoglobulin E; FeNO, Fractional Exhaled Nitric Oxide.

However, the patient’s serum total IgE remained >2500 IU/mL at each follow-up visit. We switched from omalizumab α to Dupilumab (Sanofi Winthrop Industrie) on June, and during subsequent follow-up, the efficacy of dupilumab was observed, manifested by a gradual decline in serum total IgE levels and stabilization of eosinophil counts (Table 4). The patient’s treatment course is shown in Figure 7.

**TABLE 4:** Follow-up data after switching to Dupilumab.

	10.28.2024	12.2.2024	01.06.2025	02.06.2025
FeNO (ppb)	28	29	25	20
AEC (×10^9^/L)	0.35	0.37	0.39	0.35
Serum IgE (IU/mL)	>5000	>5000	4885	4276

FeNO, fractional exhaled nitric oxide; AEC, absolute eosinophil count.

## Discussion

ABPA is an allergic inflammatory disease of the lungs caused by Aspergillus, characterized by elevated eosinophils and IgE. Clinical manifestations include cough, sputum, wheezing, and chest pain (Maturu and Agarwal, 2015a; Stevens et al., 2003). International Society for Human and Animal Mycology (ISHAM) had proposed new diagnostic criteria for ABPA in 2024 (Agarwal et al., 2024). The patient met the ISHAM diagnostic criteria for ABPA, supported by laboratory and imaging data summarized in Table 5.

**TABLE 5:** The patient met the diagnostic criteria for ABPA defined by ISHAM.

ISHAM criteria for diagnosing ABPA	Patient
Predisposing conditions (asthma, cystic fibrosis, COPD, bronchiectasis) or a compatible clinico-radiological presentation	• COPD, asthma • bronchiectasis
Essential components	
A. fumigatus-specific IgE ⩾0.35 kUA·L^−1^ Serum total IgE ⩾500 IU·mL^−1^	• A. fumigatus-specific IgE (44.4 kUA·L^−1^) • serum IgE (>2500 U/mL)
Other components (any two)	
Positive IgG against A. fumigatus Blood eosinophil count ⩾500 cells·µL−1 (could be historical) Thin-section chest computed tomography consistent with ABPA (bronchiectasis, mucus plugging and high-attenuation mucus) or fleeting opacities on chest radiograph consistent with ABPA.	• absolute eosinophil count (0.68 × 10^9^/L) • bronchiectasis, mucus plugging

A. fumigatus: Aspergillus fumigatus.

The patient had multiple established risk factors for IPA, including COPD, asthma, bronchiectasis, and prolonged oral glucocorticoids use. During the clinical course, two sequential BALF GM testing >1.0 μg/L, with concurrent Aspergillus species isolation on fungal culture. These findings fulfill the diagnostic criteria for proven IPA as current guidelines (Ullmann et al., 2018; Garcia-Vidal et al., 2019; Patterson et al., 2016a; Patterson et al., 2016b; Donnelly et al., 2020). The patient tested negative for Anti-Neutrophil Cytoplasmic Antibodies (ANCA) vasculitis markers, BALF NGS detected no nontuberculous mycobacterial (NTM) pathogen sequences, and Aspergillus IgG was also negative. We ruled out the differential diagnoses of eosinophilic granulomatosis with polyangiitis (EGPA), NTM infection, and chronic necrotizing aspergillosis (CNA), ultimately confirming the diagnosis of ABPA complicated by IPA.

The occurrence of ABPA combined with IPA is relatively rare (De Pauw et al., 2008; Tamkeviciute et al., 2024; Donnelly et al., 2020). Some researchers believe that the use of multiple broad-spectrum antibiotics has altered the local microbial flora, making Aspergillus the dominant species, especially in patients with structural lung disease and those using glucocorticoids (Chung et al., 1994). The patient was treated with itraconazole as well as oral glucocorticoids in the early stage, itraconazole can reduce the metabolism of glucocorticoids, increasing its plasma concentration and half-life. This may also serve as a potential pathogenic contributor to the development of IPA(Varis et al., 1998; Varis et al., 2000). There have been reported cases in the literature of patients with ABPA who developed IPA after treatment with glucocorticoids and itraconazole (Maturu and Agarwal, 2015b; Ganassini and Cazzadori, 1995).

The treatment plans for ABPA and IPA differ significantly, and inappropriate diagnosis and treatment can severely affect the patient’s prognosis (Brown et al., 2012). The therapeutic paradox between glucocorticoid-induced immunosuppression and antifungal demands complicates the management of ABPA complicated by IPA.

In patients with ABPA, the hypersensitivity response to Aspergillus sensitization manifests as a CD4^+^ T cell-driven Th2-mediated immune reaction. This mechanism triggers the release of interleukin (IL)-4, IL-5, IL-13, and chemokines, which induce mast cell degranulation and promote the recruitment/activation of inflammatory cells (neutrophils and eosinophils) within the airways. Concomitantly, elevated IgE production sustains a self-perpetuating Th2 inflammatory cascade, clinically characterized by peripheral blood eosinophilia, increased serum total IgE, Aspergillus-specific IgE seropositivity, and pulmonary eosinophilic infiltration (Luo et al., 2024; Moss, 2005). Multiple biologics targeting key ABPA inflammatory pathways are now clinically used, including omalizumab α, dupilumab, mepolizumab, and benralizumab, which effectively target type 2 inflammation in ABPA.

Omalizumab α ameliorates ABPA pathogenesis by neutralizing circulating IgE and preventing its binding to the high-affinity FcεRI receptor on mast cells and basophils, thereby suppressing IgE-mediated degranulation and Th2-driven airway inflammation. In a clinical randomized controlled study, omalizumab α significantly reduced the incidence of exacerbations in ABPA patients, as well as serum total IgE and FeNO levels (Voskamp et al., 2015). Several meta-analyses have also confirmed that omalizumab α can improve the lung function and asthma control in patients with ABPA, reduce the dosage of glucocorticoids, and even discontinue their use (Voskamp et al., 2015; Jin et al., 2023; Chen et al., 2024).

Guidelines from the ISHAM now endorse omalizumab α as a novel therapeutic method, providing a mechanistic rationale to resolve this dilemma (Agarwal et al., 2024). Omalizumab α can modulate allergic inflammation while circumventing the risks of generalized immunosuppression inherent to conventional glucocorticoid-based protocols. Therefore, we implemented a dual-pathway therapeutic strategy: substituting glucocorticoids with omalizumab α to mitigate ABPA-related allergic inflammation without glucocorticoid-induced immunosuppression, combined with voriconazole for IPA treatment. This strategy resolves the therapeutic paradox between ABPA and IPA management.

The therapeutic efficacy of ABPA treatment is mainly assessed through the levels of eosinophils and total serum IgE(Agarwal et al., 2024). At the 3-month follow-up after omalizumab α initiation, the patient’s serum IgE remained markedly elevated (>25,000 U/L). This persistent elevation may reflect a disequilibrium between IgE production and clearance mechanisms, as therapeutic neutralization by omalizumab α may inadequately counterbalance ongoing IgE synthesis. Dupilumab selectively inhibits the alpha subunit of the interleukin-4 (IL-4) receptor, a component shared by both IL-4 and IL-13 signaling pathways (Rabe et al., 2018). Beyond its direct inhibitory effect on IL-13-mediated signaling, this therapeutic agent further suppresses interleukin-5 (IL-5) production by modulating group 2 innate lymphoid cells (ILC2s), thereby extending its immunoregulatory impact (Patel et al., 2020). Blockade of IL-4 can inhibit B cell-mediated IgE production, while IL-13 pathway inhibition attenuates airway smooth muscle hyperreactivity and epithelial mucus hypersecretion (Walford and Doherty, 2014). The dual mechanism of dupilumab in targeting type 2 inflammation may confer therapeutic advantages in ABPA cases refractory to conventional antibody therapies, positioning it as a promising intervention for treatment-resistant ABPA phenotypes.

Dupilumab has demonstrated therapeutic efficacy in ABPA across multiple case reports, with evidence supporting its ability to suppress IL-4/IL-13-driven Th2 inflammation and reduce dependency on systemic glucocorticoids (Sumi et al., 2024; Mummler et al., 2020; Kawasaki et al., 2024; Hamakawa and Ishida, 2024; Kai et al., 2022; van der Veer et al., 2021). Although clinical evidence for dupilumab in ABPA remains limited, it may be effective in patients who are resistant to treatment with omalizumab α (Lamothe et al., 2025; Asano et al., 2025; Motohashi et al., 2025). A retrospective analysis demonstrated that anti-IL-4Rα therapy significantly reduced the frequency of acute exacerbations and oral glucocorticoid dependence in patients with ABPA compared to anti-IgE therapy, likely attributable to its dual blockade of IL-4/IL-13 signaling that disrupts the Th2-inflammatory cascade driving both IgE production and eosinophilic airway injury (Lamothe et al., 2025).

Both univariable and multivariable analyses demonstrated that patients failing to attain clinical remission exhibited significantly elevated serum total IgE levels (Tanaka et al., 2025), with those presenting elevated total IgE levels showing increased likelihood of disease exacerbation (Oguma et al., 2018). In this case, ABPA remission following the use of omalizumab α was temporary. There are very few studies on the use of mepolizumab and benralizumab in ABPA, and they are not included in our considerations. Therefore, given the patient’s refractory elevation of serum IgE levels despite optimized omalizumab α therapy, we transitioned to dupilumab to target upstream IL-4 receptor α subunit signaling—a strategic shift addressing persistent Th2-driven IgE overproduction. Subsequent follow-up results after switching to dupilumab also demonstrated clinical efficacy, which aligned with our expectations and confirmed the mechanistic advantages of dupilumab over omalizumab α.

This study has several limitations inherent to its single-case report, including the absence of long-term follow-up data. While this case cannot definitively establish the efficacy of dupilumab combined with antifungal agents in ABPA-IPA comorbidity, it provides theoretical basis for future rigorously designed randomized controlled trials evaluating targeted immunomodulatory strategies in this high-risk population. Future randomized controlled trials comparing omalizumab α and dupilumab in ABPA-IPA comorbidity are warranted to delineate their roles in mitigating allergic and invasive disease trajectories while preserving antifungal immunity.

## Conclusion

In summary, IPA represents a rare yet life-threatening complication in patients with ABPA. Early screening for IPA is critical in this population, particularly when ABPA manifests with refractory symptoms or atypical radiographic features. Notably, biologic agents such as omalizumab α and dupilumab offer a advantage, they mitigate ABPA-driven Th2 inflammation while reducing reliance on systemic glucocorticoids. Therapeutic regimens should prioritize glucocorticoid-sparing strategies through targeted biologics combined with mold-active triazoles (e.g., voriconazole), aiming to concurrently control allergic exacerbations, prevent bronchial remodeling, and minimize immunosuppression-related risks. This treatment plan shift emphasizes the importance of precision immunomodulation to break the vicious cycle of fungal sensitization and invasive progression in ABPA-IPA comorbidities.

## Data Availability

The original contributions presented in the study are included in the article. Further inquiries can be directed to the corresponding author.
